# A Snapshot of COVID-19 Vaccine Discourse Related to Ethnic Minority Communities in the United Kingdom Between January and April 2022: Mixed Methods Analysis

**DOI:** 10.2196/51152

**Published:** 2024-03-26

**Authors:** Nazifa Ullah, Sam Martin, Shoba Poduval

**Affiliations:** 1 Research Department of Primary Care & Population Health University College London London United Kingdom; 2 Vaccines and Society Unit, Oxford Vaccine Group University of Oxford Oxford United Kingdom; 3 Institute of Health Informatics University College London London United Kingdom

**Keywords:** COVID-19, ethnic minorities, vaccine, hesitancy, social media, discourse, minority groups

## Abstract

**Background:**

Existing literature highlights the role of social media as a key source of information for the public during the COVID-19 pandemic and its influence on vaccination attempts. Yet there is little research exploring its role in the public discourse specifically among ethnic minority communities, who have the highest rates of vaccine hesitancy (delay or refusal of vaccination despite availability of services).

**Objective:**

This study aims to understand the discourse related to minority communities on social media platforms Twitter and YouTube.

**Methods:**

Social media data from the United Kingdom was extracted from Twitter and YouTube using the software Netlytics and YouTube Data Tools to provide a “snapshot” of the discourse between January and April 2022. A mixed method approach was used where qualitative data were contextualized into codes. Network analysis was applied to provide insight into the most frequent and weighted keywords and topics of conversations.

**Results:**

A total of 260 tweets and 156 comments from 4 YouTube videos were included in our analysis. Our data suggests that the most popular topics of conversation during the period sampled were related to communication strategies adopted during the booster vaccine rollout. These were noted to be divisive in nature and linked to wider conversations around racism and historical mistrust toward institutions.

**Conclusions:**

Our study suggests a shift in narrative from concerns about the COVID-19 vaccine itself, toward the strategies used in vaccination implementation, in particular the targeting of ethnic minority groups through vaccination campaigns. The implications for public health communication during crisis management in a pandemic context include acknowledging wider experiences of discrimination when addressing ethnic minority communities.

## Introduction

In December 2020, the United Kingdom began its COVID-19 vaccination program [1]. Population-level studies have demonstrated persistently lower levels of uptake of the first dose of the COVID-19 vaccine among all minority groups compared with the White British ethnic group, and these differences widened over time [2]. Vaccine hesitancy, that is, the delay or refusal of vaccinations despite the availability of services [3], has been recognized as a significant contributing factor to the unequal uptake of COVID-19 vaccines across different ethnic groups [4]. This contrasts with anti-vaxxers, defined as a group of individuals who may refuse most if not all vaccinations [5].

Several studies have investigated the causes of hesitancy within the context of the COVID-19 vaccine [6-8]. A summary provided by the World Health Organization 3 C’s model identifies the following 3 core areas that influence hesitancy: confidence (toward the vaccine or its provider), complacency (perceived risk toward vaccine-preventable diseases), and convenience (access to vaccines) [8].

Historical mistrust toward governments, racism within the health care system, and lack of diversity in medical research are further points in the literature noted as contributing factors to hesitancy specifically in relation to ethnic minority groups [5,9-11].

Among the many factors noted, the rising use of social media as an interactive health ecosystem fuelling vaccine hesitancy remains a recurrent concern contributing to vaccine confidence, as identified in the literature [12]. During the COVID-19 pandemic, there was noted to be the issue of an “infodemic,” that is, the spread of large volumes of information including false or misleading information [13,14]. Social media played a significant role in this infodemic via the rapid dissemination of information. A total of 49% (n=980) of respondents to an online survey in the United Kingdom used social media as a source of information about COVID-19 [15] and Twitter reported COVID-19–related posts every 45 milliseconds in 2020 [16-18].

Social media was a prominent source of misinformation during the pandemic, particularly in relation to vaccinations. Misinformation refers to false or inaccurate information shared unknowingly and without the intention to cause harm [19]. Studies have highlighted the correlation between exposure to misinformation on social media and both vaccine hesitancy and reduced compliance to public health measures and disease prevention activities such as vaccinations [20-22]. Loomba et al [21] conducted a randomized control trial in the United Kingdom and the United States quantifying exposure to misinformation related to COVID-19 vaccines on social media platforms and vaccination intent and found that exposure to misinformation resulted in a 6.2% decline in the number of respondents who would “definitely” take the vaccine in comparison to control groups. Betsch et al [20] found that even brief exposure to vaccine-critical websites would increase an individual’s overall perception of vaccine risk in comparison to exposure to control websites. Furthermore, negative information related to vaccinations tends to encourage greater user engagement in comparison to positive content [23,24]. This may be due to the persuasive narratives and powerful imagery often used in antivaccination content [22]. Another important note is the creation of “echo chambers” on social media, that is, the network of users in which each user encounters beliefs supporting pre-existing opinions without being exposed to opposing viewpoints [12]. Such effects further reinforce antivaccine perspectives by connecting like-minded individuals thereby amplifying antivaccine narratives and potentially dampening vaccination attempts [7].

Despite the clear role of social media as a source of information during a pandemic and its impact on hesitancy and consequently vaccination attempts, there is little published data on its role in the public discourse on COVID-19 vaccines among ethnic minority groups in the United Kingdom. For ethnic minority communities, there may be a greater reliance on social media as a source of information because of barriers in accessing health care and health information due to, for example, language barriers or poor health literacy [25]. Dickson et al [26] study looking at culturally and linguistically diverse communities found that individuals would often receive information by “word of mouth,” that is, from peers from the same cultural groups as these would be seen as a trusted source of information. Those peers themselves would receive information from social media platforms such as Facebook and WhatsApp [26]. It is for these reasons that understanding hesitancy in minority communities through the lens of social media is of paramount importance.

Our article aims to address the following research question by obtaining data from social platforms Twitter and YouTube between January and April 2022:

1. What was the discourse related to and within ethnic minority communities on social media platforms?
2. What were the general sentiments and stances of social media posts analyzed?
3. What were the most frequent topics of conversation seen on social media?

## Methods

### Study Design

The study analyzed Twitter and YouTube comments using a mixed method approach. This approach is often used when analyzing social media posts due to the large quantity of data that allows for both quantitative exploration as well as qualitative analysis of the contents, further enhancing the understanding of a research topic [27].

### Data Source

We obtained publicly available data from Twitter and YouTube comments in the United Kingdom between January 17, 2022, and April 7, 2022, using the software Netlytics (for Twitter posts) and YouTube Data Tools (for YouTube data) [28,29]. The platforms Twitter and YouTube were selected because they provided the most openly available application programming interfaces (APIs) [16]. APIs are mechanisms that enable 2 software components to communicate with one another and allow third-party developers to access data such as tweets and YouTube comments [30].

Drawing on prior research studies on social media and the COVID-19 vaccine [6-8], a pilot Boolean search strategy was created (Multimedia Appendix 1) and applied to Netlytics and YouTube Data Tools in order to understand recurrent topics of discussion around the vaccine. As of April 7, 2022, which was the end date for our data retrieval, the UK government was in the midst of its booster vaccination rollout and proposed that all eligible adults older than 18 years would be offered the booster vaccine [31]. The government had also proposed mandatory vaccinations for National Health Service workers during this period, a proposal that was later reversed by January 31 [31,32].

Consequently, our preliminary search of the data informed the development of a second more focused Boolean search (Multimedia Appendix 1) containing keywords and relevant hashtags such as those related to booster vaccination campaigns. All posts were screened by NU according to our inclusion and exclusion criteria (Textbox 1) and duplicates were removed. To note, the platform Netlytics provided geo-coded social media data, enabling us to ensure that tweets analyzed are from those based in the United Kingdom [28]. Geo-coded data may be provided through the Netlytics filtering system that allows users to include or exclude tweets based on a given radius and latitude [28]. The YouTube videos selected were checked by NU to ensure the content was related to the United Kingdom.

A total of 260 tweets (that were retweeted 15,331 times) and 156 comments from 4 YouTube videos remained after duplications were removed and went through the inclusion and exclusion processes.

Data were managed on Microsoft Excel [33]. To meet ethics and European Union General Data Protection Regulation guidelines [34,35], usernames were anonymized using a Python script [36] to replace all usernames and links with an encrypted tag code.

**Inclusion and exclusion criteria.**

**Inclusion criteria**

- Posts where ethnic minority groups are discussed or mentioned (all ethnic groups except White British groups). For example use of the terms: (“BAME” OR “BME” OR “Black*” OR “Asian*” OR “minority ethnic” OR “ethnic minority” OR “minority” OR “non-white” OR “raci*” OR “MinorityHealth” OR “Minority” OR “race”; see Multimedia Appendix 1 for full list).
- Posts referring specifically to COVID-19 vaccination. For example use of the terms: (“vaccin*” OR “immunis*” OR “vax*” OR “jab*” OR “covidvaccin*” OR “COVIDVaccine” OR “COVID19” OR “COVID19Vaccine” OR “CovidVaccine” OR “covid-19” OR “covid vaccine,” OR “covid vax”).
- Example of a hypothetical post to include: “Racism and historical injustices fuelled low COVID vaccine uptake by minorities.” We would include this as it refers specifically to COVID-19 vaccinations in relation to ethnic minority groups.

**Exclusion criteria**

- Posts that discussed the COVID-19 vaccine without mention of ethnic minority groups.
- Posts that do not mention the COVID-19 vaccine.
- Posts by individuals not based in the United Kingdom.
- Example of hypothetical post to exclude: “COVID has caused many deaths.” This would be excluded as there is no mention of the COVID-19 vaccines and minority groups.

### Measures of Variables

#### Themes

In order to identify key themes of topics in our social media posts, we developed an initial analytic coding framework (Multimedia Appendix 1) that was informed by previous literature investigating causes of hesitancy as discussed previously [6-11]. We added the coding framework to a Microsoft Excel spreadsheet, with codes in the columns and social media posts entered as individual comments in the rows. The framework was refined during team discussions and all authors applied the same framework to the data.

#### Sentiment and Stance Coding

In order to appreciate the nuanced opinions on social media and how they may be contributing to positive or negative attitudes toward COVID-19 vaccines [37], individual posts were manually coded into sentiment and stance categories (Multimedia Appendix 1). Sentiment refers to the overall tone of a post and the categorization of opinions toward a subject expressed by the author (being positive, negative, neutral, or ambiguous) [37,38]. Whereas stance refers to the process of determining the author’s attitude or stance toward a target [37]. For example, a post may have a positive sentiment toward anti-vaxxers but a negative stance toward vaccines.

Our stance framework (Multimedia Appendix 1) was informed by the Leask et al [39] categorization of vaccine intention. Hypothetical examples derived from our social media data and the sentiment and stance assigned to each text can be found in Multimedia Appendix 1. We coded the sentiment and stance of the data and added both to our Microsoft Excel spreadsheet. For example, a positive sentiment post would be coded as P and a positive stance post would be coded PS (codes clarified in Multimedia Appendix 1). Any disagreements regarding the classification of the social media posts were discussed during team meetings where a unanimous decision was made regarding their categorization.

#### Data Analysis Procedure

The percentage of Twitter and YouTube posts assigned to each sentiment and stance category was calculated and posts coded according to themes derived were quantified for further analysis. Where a post was assigned more than 1 theme, they were calculated as separate entities, for example, if a post was assigned to themes 1 and 2, they would be included separately when calculating overall percentages.

Taking an inductive approach, we collated the results from our analysis and a random sample of posts (30 tweets and 20 YouTube comments) were selected for a more detailed analysis and qualitative interrogation of the data to address our overall aims [40,41]. Our thematic analysis framework was updated accordingly to ensure themes identified were grounded in the data obtained.

#### Network Analysis and Visualization

Network analysis was also carried out to highlight the most prevalent topics of discussion on social media. Text network analysis software Infranodus (Nodus Labs) was used [42]. Data were imported into Infrandous and semantic networks were generated with data organized by specific topics and subtopics. Word clusters were derived using modularity measurement tools to highlight the most common topics of conversations within a group of tweets or YouTube comments. Keywords obtained using betweenness centrality, an analysis of connections between subtopics or words that link different clusters of conversations together, helped deepen our understanding of influential words or topics that may be linking clusters of conversations together. Word frequency analysis was then performed to highlight the most frequent and weighted subtopics of conversations.

### Ethical Considerations

Ethics and data protection approval were obtained from University College London’s (UCL’s) Research Ethics Committee (ID 21773/001) and UCL Data Protection Officer (registration number: Z6364106/2021/10/60). All data obtained were publicly available via YouTube’s and Twitter’s APIs.

## Results

### Overview

Using our search strategy, a total of 44,144 Twitter posts and 9 YouTube videos were identified. After the deduplication process and screening according to inclusion and exclusion criteria, a total of 416 deduplicated social media posts were identified. This included 260 tweets (that were retweeted 15,331 times) and 156 comments from 4 YouTube videos that were included in our analysis. Video 1 was a documentary exploring whether ethnic minorities and in particular minority health care workers, should be prioritized in having the COVID-19 vaccine. Video 2 was a debate on a news channel looking at the causes of vaccine hesitancy specifically in minority communities. Videos 3 and 4 were documentaries that focused on similar topics and explored the causes of vaccine hesitancy within ethnic minority communities. Out of the 4 videos analyzed, video 2 had the highest number of total comments of 1273.

### Themes

The following 3 themes were identified in the discourse on both Twitter and YouTube: concerns related to vaccination implementation, questions over vaccine development and safety, and wider systemic issues such as institutional racism and historical abuse of power.

Concerns related to vaccine implementation were mostly related to mandatory vaccinations for health care workers and the wider public and public health messaging, in particular the use of the phrase “BAME.” Most of the topics connected to vaccine development were related to the speed of development of the vaccine and its side effects. Comments alluding to conspiracy theories suggesting the vaccine to be gene-altering, experimental in nature, and causing infertility as a side effect were particularly seen on YouTube. Moreover, systemic racism, that is, a form of racism that is embedded within the laws and regulations of a society [43], was a prevalent topic noted under the theme of wider systems. The topic of racism was particularly prevalent when discussing the strategies of vaccine rollout such as mandatory vaccinations for health care workers and the focus on vaccinating ethnic minorities. Table 1 summarizes the themes noted in our discourse analysis with hypothetical examples of tweets and YouTube comments derived from our data. Hypothetical examples are given in order to abide by ethics guidelines preventing any identifiable information from being published.

**Table 1:** Themes and topics from COVID-19 vaccine discourse between January and April 2022 on Twitter and YouTube in the United Kingdom.

Themes or topics of discussion	Examples of tweets	Example of YouTube comments
Vaccination implementation	“There is a witch hunt against black and Asian NHS workers who don’t want the vaccine” “We need to stop this race baiting by focusing all the time on BAME”	“Why are we obsessing over BAME groups. It’s just another form of segregation and racism.”
Vaccine development and safety	“The vaccine is an experiment aimed at wiping out humanity” “Stay away from the jab, it’s likely to cause autism in Black people” “You’re less likely to die from having the vaccine then from Covid- vaccines undergo a lot of testing to rule out long term side effects”	“The vaccine is just part of a scheme to reduce the population of minorities. Look at the testing they have done on African women causing infertility” “The vaccine isn’t safe, it was made too quickly”
Wider systemic issues	“It’s not just about mistrust towards institutions but health racism is a real issue too.”	“Remember the Tuskegee trials? They are just trying to use Black and Asian people as guinea pigs now”

### Sentiment and Stance

Table 2 demonstrates the sentiment and stance of tweets and YouTube comments as a percentage. In general, YouTube depicted a higher percentage of negative sentiment and stance posts over Twitter.

**Table 2:** Percentage of Twitter and YouTube posts in the United Kingdom and their sentiment and stance.

Characteristic		Twitter, n (%)	YouTube, n (%)
**Sentiment**			
	Positive	61 (23.5)	12 (7.7)
	Negative	48 (18.5)	49 (31.4)
	Ambiguous	21 (8.1)	16 (10.3)
	Neutral	130 (50)	79 (50.6)
**Stance**			
	Positive	69 (26.5)	18 (11.5)
	Negative	37 (14.2)	48 (30.8)
	Ambiguous	45 (17.3)	74 (47.4)
	Neutral	109 (41.9)	16 (10.3)

### Network Analysis

Network analysis identified the following 4 key most frequent topics of conversations on Twitter, portrayed as clusters of tweets: booster vaccinations (cluster 1), mistrust (cluster 2), vaccination risk (cluster 3), and racism (cluster 4). Cluster 1 was the most popular group of conversations, with 23% (n=60) of tweets containing the keywords “vaccine,” “covid,” and “uptake.” Most tweets were related to encouraging booster vaccinations in minority communities. Some comments were noted addressing issues around misinformation and hesitancy in an endeavor to encourage booster vaccinations. Cluster 2 of tweets contained keywords, “trust,” “pandemic,” and “work.” There were discussions related to vaccinations for health workers as well as topics related to health inequalities in minority communities and historical abuses of power. Cluster 3 contained the keywords “jab,” “risk,” and “explain,” with conversations focused on the side effects of vaccines. Cluster 4 contained keywords “black,” “people,” and “race,” paying particular attention to the uptake of vaccines within the Black African and Caribbean community.

The word “vaccine” had the greatest betweenness centrality of 0.71, meaning that it was this phrase that predominantly linked the different clusters of conversations in the network, that is, the distribution of tweets, together. This is followed by “minority,” “covid,” and then “black.” The network structure, that is, the network of comments that people have made around a specific topic and their relation to one another, had a focused influence distribution of 80% with a modularity measure of 0.28. This illustrates that although there were several conversations related to the vaccine, the conversations were focused on a small number of key themes discussed above. Further statistics discussing the average degree and weighted betweenness between nodes can be found in the Multimedia Appendix 2. Further images of individual clusters can be found in Multimedia Appendix 3.

Regarding YouTube, video 1 keywords found in the most popular cluster of conversations included “race,” “worker,” and “baiting.” “Worker,” in this context was related to debates on whether ethnic minority health care workers should be prioritized in having the vaccine. Most conversations were hence related to racial tension and worries that vaccine campaigns prioritizing specific ethnic minority communities would seed further division. Video 2 had a similar focus in that the main topic of conversation was related to critiquing the vaccination program and the focus on ethnic minorities. Both videos 1 and 2 had a focused network structure reflecting that opinions expressed were similar in nature, hence a focused structure.

Video 3 had the most popular cluster of conversations containing the keywords “time,” “treatment,” and “American.” This video had multiple mentions of the Tuskegee trial, an experiment from 1932 to 1972 that involved the unethical testing of over 400 Black men who were falsely led to believe that they were being treated for syphilis [44]. Video 4 contained similar clusters of conversations related to mistrust. Both videos had a diverse network structure due to the various causes of vaccine hesitancy discussed in the comments sections. Multimedia Appendix 4 provides examples of conversation clusters in Videos 1 and 3 related to themes of racism and mistrust.

## Discussion

### Principal Findings

Our study aimed to provide an overview of the discourse related to and within ethnic minority communities on the social media platforms Twitter and YouTube. This is one of the few studies to our knowledge that has explored the use of social media in this manner specifically related to ethnic minority communities. The summary of our results can be found in Table 3. The themes identified were intrinsically linked with one another. For example, concerns raised around vaccination strategy such as the use of the term “BAME” were associated with themes related to wider systemic issues such as racism. The higher percentage of negative sentiment and stance seen on YouTube may be attributed to the fact that on YouTube, there is less of a focus on the individual profile page giving rise to the perception of greater anonymity when posting negative statements [45]. However, the nature of the YouTube videos may have also contributed to the higher levels of negative posts. For example, video 1 explored the potential public health policy of prioritizing minority groups for vaccination, a controversial debate that was not mentioned in the Twitter data we had collected. Naturally, with such contentious issues, greater negative comments could be seen as users shared their opinions via the comment section.

**Table 3:** Summary of results^a^.

Codes grouped to form themes and topics		Percentage of Twitter posts assigned, n (%)	Percentage of YouTube posts assigned, n (%)	Keywords from Twitter and YouTube obtained from network analysis
**Vaccine implementation**				
	Booster vaccinations Communication strategies Control measures, for example, mandatory vaccinations	301 (60.2)	110 (44.9)	Vaccine, COVID, and uptake
**Vaccine development and side effects**				
	Speed of development Side effects	15 (3)	18 (5.3)	Jab, risk, and explain
**Wider systems**				
	Systemic racism Mistrust toward institutions Historical abuses of power	184 (36.7)	122 (49.8)	Black, people, race, trust, pandemic, work, worker, and baiting

^a^Where a post was assigned more than 1 theme, they were calculated as separate entities, for example, if a post was assigned to themes 1 and 2, they would be included separately when calculating overall percentages.

Our paper contributes to the existing literature by highlighting that one of the predominant and most popular topics of conversations within ethnic minority communities during the 3 months of data analyzed, was related to the implementation of the vaccination program, particularly communication strategies adopted during part of the booster vaccine rollout. The focus on ethnic minority communities and alienation of these groups through terms such as “BAME,” in vaccination campaigns, has been highlighted from our data as being divisive and causing further racial division. Policies such as mandatory vaccinations for health workers were deemed as particularly discriminatory toward minority workers. In fact, it seemed to have further propelled discussions related to trust or mistrust toward institutions on social media. Few papers explicitly highlight this connection between vaccination implementation strategies propelling concerns related to racism and fuelling further mistrust toward institutions specifically within minority communities.

There is an acknowledgment in the literature about the importance of adopting appropriate communication strategies in public health messaging. The Commission on Race and Ethnic Disparities has acknowledged the generalization that terms such as “BAME,” can cause [46]. Consequently, recent government guidelines have recommended using the terms “ethnic minorities” or “people from ethnic minority backgrounds” in communication [46]. Coccia’s [47] study looking at the maximum level of COVID-19 vaccinations that could be achieved across 150 countries without social impositions, for example, restrictions on public gatherings and government lockdowns, found the maximum level to be 70% based on normal hesitancy in society. In order to increase the number of vaccinated above this threshold, communicating effectively using “humble inquiry, compassionate listening and storytelling,” is a more effective approach than implementing strict health policies that impact individual freedoms [47].

Our study depicted how opinions toward vaccination strategies were linked to concepts related to trust and mistrust toward institutions. Mistrust toward institutions contributing to hesitancy in minority communities has already been noted by previous studies [5,7]. A systematic review looking at factors influencing vaccination uptake in minority communities found 6 studies that attributed lower uptake to “mistrust including pre-existing lower scientific or medical trust, conspiracy suspicions and attitudes” [48].

Moreover, our paper suggested that mistrust was mostly directed toward governments and pharmaceutical companies. These findings are echoed in a study looking at survey data from 100 countries and the roles of different forms of trust in predicting vaccine hesitancy [49]. Trust in political institutions was a consistent predictor of vaccine hesitancy [49]. The impact of a lack of trust in governments in vaccination attempts is exemplified in Romania’s COVID-19 vaccination strategies. Romania has one of the lowest confidence rates in their national government and this played a significant role in the low COVID-19 vaccination rates seen [50]. Analysis of comments from Romania’s #storiesfromvaccination campaign, found politically independent sources, for example, from health experts and laypersons, were deemed as being legitimate sources of information and consequently more trustworthy [50].

The continued concerns over vaccine development and side effects identified in our data are consistent with other studies investigating the causes of hesitancy in ethnic minority communities [11]. Fertility was a side effect most often noted to be of concern on YouTube and this is further supported by existing literature. A qualitative study of 12 participants done in 2021 exploring vaccine hesitancy in ethnic minority communities found infertility as a prevalent theme, as well as concerns related to period irregularities and breastfeeding [4]. Conspiracy theories suggesting the vaccine to be gene-altering or experimental in nature were also reported in our results. The spread of such theories could have a detrimental effect on vaccination efforts as studies have suggested that vaccination conspiracy belief was the most relevant predictor in willingness to get vaccinated (ie, a negative association) [51,52].

### Strengths and Limitations

The findings of our paper must be considered in light of the limitations of our study. First, our data extraction was limited by Twitter and YouTube API policies that restrict the amount of data that can be extracted over a certain period of time [28,29]. Moreover, our data set was small with a total of 416 social media posts analyzed. However, a rigorous qualitative analysis was conducted with multiple steps taken to validate our analysis as described in the methodology. Furthermore, the demographic of individuals using Twitter and YouTube tends to represent a younger and more politically engaged population [53]. Consequently, the data obtained may be biased toward this population. However, there are few studies looking at social media as data and by capturing the opinions of a younger population, a greater understanding may be gained of the viewpoints behind a traditionally low uptake group [7]. Moreover, it is difficult to be conclusive about our sentiment and stance analysis since we were limited by looking at each individual tweet and YouTube comment as isolated posts rather than in the context in which the comment was made (ie, as part of a thread of posts). This was to ensure our methodology of analysis remained consistent and prevented us from making assumptions about the users’ beliefs or opinions.

Much of the literature looking at social media data tends to focus on Twitter, although few on YouTube, due to the ease of accessing Twitter data when compared with other social media platforms [37]. Hence, we acknowledge that this study is not representative of all social media activity on this topic. Data from other social media sites such as Facebook, Instagram, and TikTok, remain underexplored, and more research is needed looking at such platforms and their contribution to vaccine hesitancy.

### Implications and Future Directions

Our findings suggest that when planning communication strategies for future public health interventions such as vaccination, policymakers and health practitioners must acknowledge the wider experiences of individuals, particularly in minority groups. Public health messaging around the COVID-19 vaccine has placed the emphasis on ethnic minorities to become less hesitant and more trusting rather than acknowledging the systemic racism and experiences of discrimination raised by individuals [54]. Future vaccination strategies and public health messages targeted toward minority groups must make greater concerted efforts to acknowledge the historical abuses of power and contextualize hesitancy accordingly [43,54].

The sources in which information is disseminated should also be considered.

Minority communities may have a greater trust in information obtained from peers from similar cultural groups [26] and trusted community sources [55]. Hence, it is critical that communication strategies are not only culturally sensitive and tailored toward individual groups but also use trusted sources of information.

Using existing social networks would also be useful in framing vaccinations as a social norm. Descriptive norms, aka what other people do, say, and believe, have an impact on an individual’s intentions to accept a vaccine [56]. People often underestimate vaccine acceptance by others making hesitancy more noticeable and influencing their decision-making [56]. However, presenting vaccinations as a descriptive norm and correcting people’s overestimation of the prevalence of vaccine hesitancy, may have a positive impact on improving uptake [56].

When deciding what messages to communicate, having an understanding of the discourse on social media in “real time” may help guide communication strategies. By identifying trends in opinions, changes in sentiments, and stances toward a particular intervention such as vaccinations, behaviors can be anticipated [57]. Policymakers may then be able to intervene in a timely manner to encourage and sustain support [58].

What is clear to see is the importance of effective communication that explains the reasons for targeted approaches toward vaccinations in the context of a pandemic in a manner that is sensitive and addresses misconceptions from minority communities [59]. Underlying this is the need for strong governance led by effective leadership that engages communities and adjusts to a population’s needs [60,61]. Good governance not only allows for timely and effective vaccination campaigns [60-62] but also greater investment in research and development and higher public spending [60]. Greater public spending may address some of the wider socioeconomic factors that contribute to reduced vaccine uptake in minority communities [7]. More investment in research may allow governments to better address and implement nonpharmaceutical methods of control during a pandemic such as effective contract systems and stronger early warning systems [63]. Such steps will help improve governments’ prevention and preparedness for future pandemic threats.

### Conclusions

Our study highlighted the concerns minority groups had in relation to the vaccine implementation, specifically the targeting of minority groups through vaccination campaigns. The shortfalls in the communication strategies adopted to relay public health information to minority groups during the pandemic must be acknowledged if any meaningful improvement is to be made moving forward to strengthen future interventions targeted toward these groups.

## Appendix

Multimedia Appendix 1 Initial Boolean search term, final Boolean search term, thematic analysis framework, sentiment analysis coding, stance analysis coding, hypothetical examples of social media posts, and their sentiment and stance.

Multimedia Appendix 2 Statistics from network analysis.

Multimedia Appendix 3 Most frequent clusters of tweets and key themes noted in relation to the COVID-19 vaccines between Jan 2022 to April 2022 in the UK obtained from network analysis.

Multimedia Appendix 4 Conversation clusters in YouTube videos 1 and 3 related to themes of racism and mistrust in the United Kingdom.

## Abbreviations

API: application programming interface
UCL: University College London

## Data Availability

The data sets generated or analyzed during this study are not publicly available due to restrictions from the UCL Research Ethics Council that prohibit the publication of any identifiable information from social media platforms.
