# Supplementary material for: A Snapshot of COVID-19 Vaccine Discourse Related to Ethnic Minority Communities in the United Kingdom Between January and April 2022: Mixed Methods Analysis
Source: JMIR Form Res. 2024 Mar 26;8:e51152. doi: 10.2196/51152 (PMC10968668; doi:10.2196/51152)
Supplement: Multimedia Appendix 1 [file formative_v8i1e51152_app1.pdf]

## Appendix A: Initial Boolean search term with focus on BAME related keywords and hashtags

("vaccin*" OR "immunis*" OR "vax*" OR "jab*" OR "covidvaccin*" OR "covidvax*" OR "covid vaccin*" OR "coronavirusvaccin*" OR "coronavirus vaccin*" OR "coronavax*" OR "coronavacc*" OR "COVIDVaccine" OR "COVID19" OR "COVID19Vaccine" OR "CovidVaccine" OR "COVID-19") AND ("BAME" OR "BME" OR "Black*" OR "Asian*" OR "minority ethnic" OR "ethnic minority" OR "minority" OR "non-white") AND ("hesitancy*" OR "worry*" OR "refuse*" OR "uncer*" OR "exclu*" OR "uneq*" OR “discri*” OR “inequ*” OR “vulnerab*” OR “mistrust” OR "access*" OR “deprived” OR “dispropor*” OR “aggres*” OR “liberty” OR “system*” OR “infring*” OR “opposed” OR “burden” OR “uptake” OR "BringDownBarriers" OR "GetTheJab" OR "healthinequality" OR "VaccineReady" OR "raci*" OR "MinorityHealth" OR "Minority" OR "race" OR "healthequity" OR "TakeTheVaccine" OR “GetVaccinated” OR “GetBoosted” OR “GetBoostedNow”)

## Appendix B: Final Boolean search term with focus on the most relevant BAME related keywords and hashtags

("vaccin*" OR "immunis*" OR "vax*" OR "jab*" OR "covidvaccin*" OR "COVIDVaccine" OR "COVID19" OR "COVID19Vaccine" OR "CovidVaccine" OR "COVID-19") AND ("BAME" OR "BME" OR "Black*" OR "Asian*" OR "minority ethnic" OR "ethnic minority" OR "minority" OR "non-white") AND ("hesitancy" OR "worry" OR "refuse" OR "uncer*" OR "exclu*" OR "uneq*" OR “discri*” OR “inequ*” OR “vulnerab*” OR “mistrust” OR "access" OR “deprived” OR “dispropor*” OR “aggres*” OR “liberty” OR “infring*” OR “opposed” OR “burden” OR “uptake” OR "raci*" OR "MinorityHealth" OR "Minority" OR "race" OR

"healthequity" OR "TakeTheVaccine" OR “GetVaccinated” OR “GetBoosted” OR “GetBoostedNow” OR "omicron” OR “booster” OR “preg*” OR “maternal")

## Appendix C: Thematic analysis framework

1. **Individual concerns**
    - Individual freedom
        - Vaccines mandates and passports
        - Individual liberties
    - Concerns regarding the actual vaccine
        - Safety, side effects, efficacy and necessity of vaccine
        - Concerns over vaccine development
    - Individual perception of risk

2. **Community**
    - Convenience
        - Access to centres - including location of vaccine delivery, relative cost, time and distance to access vaccine
    - Necessity
    - Altruistic motivations
    - Cultural barriers

3. **Institutions**
    - Trust

    - Mistrust toward news outlets: Unclear and unreliable data, fake news, complex information, conflicting vaccine information, changing guidance, and contraindication of information between different sources
    - Mistrust towards institutions e.g. governments, pharmaceutical companies and healthcare systems

4. **Wider systems**
    - Racism
        - Systemic racism
        - Historical marginalisation and disempowerment
    - Health inequity and inequality
        - Inequity in vaccine management, distribution, access and quality
    - Representation
        - Diversity in healthcare research
        - Representation of different ethnic groups in vaccine campaigns

## Appendix D: Sentiment analysis coding

**Ambiguous (A)**

- Content contains indecision, uncertainty on the risks or benefits of the vaccine
- Post contains both approving and disapproving information

**Positive (P)**

- Post communicates public health benefits or safety of vaccination
- Post encourages uptake of the vaccine
- Post describes the risk of not taking the vaccine
- Post tackles negative misconceptions of the vaccine

**Negative (N)**

- Post contains negative attitude towards the vaccine (e.g. safety issues, necessity, effectiveness and side effects)
- Post discourages others to take the vaccine

**Neutral (NT)**

- Post contains no elements of uncertainty, promotional or negative content
- Post contain general statement or links to items e.g. news articles, with no expression on sentiment
- Post includes factual statements/recommendations about the vaccine but no other sentiment

## Appendix E: Stance analysis coding

**Ambiguous stance (AS)**

- Cautious acceptors: Those with minor concerns about vaccination risks but still vaccinate.
- The hesitant: Those who have significant concerns about vaccination and are focused on vaccine risks rather than benefits.
- Late or selective vaccinator: Those who have significant doubts and concerns about vaccines and may opt out of many vaccinations.

**Positive stance (PS)**

- Unquestioning acceptors: Those with no questions or concerns about the safety of vaccines or the need to vaccinate.

**Negative stance (NS)**

- Those who refuse most if not all vaccinations

**Neutral Stance (NTS)**

- Those whose stance remain unclear - neither cautious acceptor, hesitant, late or selective vaccination, unquestioning or refuser.

## Appendix F: Hypothetical examples of social media posts and their sentiment and stance

| Text | Sentiment (subject) | Stance (target) |
|---|---|---|
| "We need to encourage Black and ethnic minorities to get vaccinated- vaccines save lives." | Positive (vaccines) | Positive (vaccines) |
| "Antivaxxers tell the truth." | Positive (antivaxxers) | Negative (vaccines) |
| "Forcing vaccinations on people is a violation of their human rights." | Negative (vaccination policy) | Ambiguous stance (vaccine) |
| "There are large disparities in uptake of the Covid-19 vaccine amongst minority groups in comparison to the White British population." | Neutral (vaccine) | Neutral (vaccine) |
