# Supplementary material for: A Snapshot of COVID-19 Vaccine Discourse Related to Ethnic Minority Communities in the United Kingdom Between January and April 2022: Mixed Methods Analysis
Source: JMIR Form Res. 2024 Mar 26;8:e51152. doi: 10.2196/51152 (PMC10968668; doi:10.2196/51152)
Supplement: Multimedia Appendix 3 [file formative_v8i1e51152_app3.pdf]

Most frequent clusters of tweets and key themes noted in relation to the Covid-19 vaccines between Jan 2022 to April 2022 in the UK obtained from Network Analysis

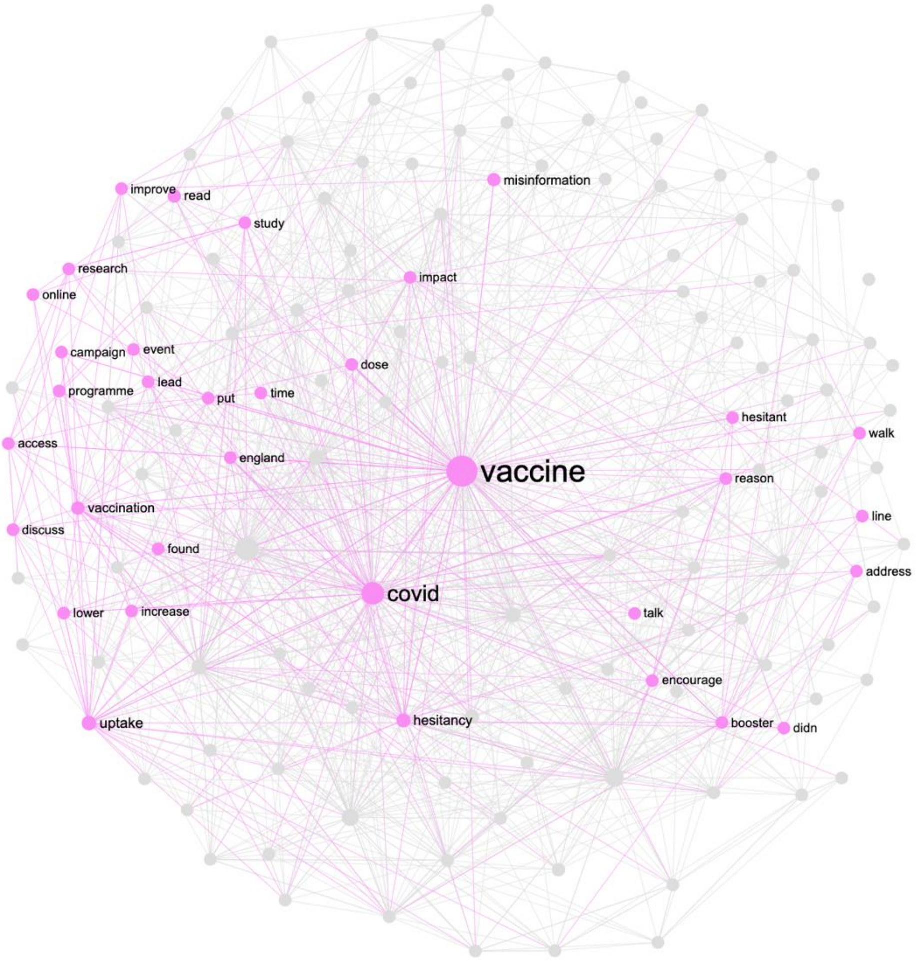

Cluster 1: Vaccine uptake in the community
Keywords: "vaccine," "covid," and "uptake"

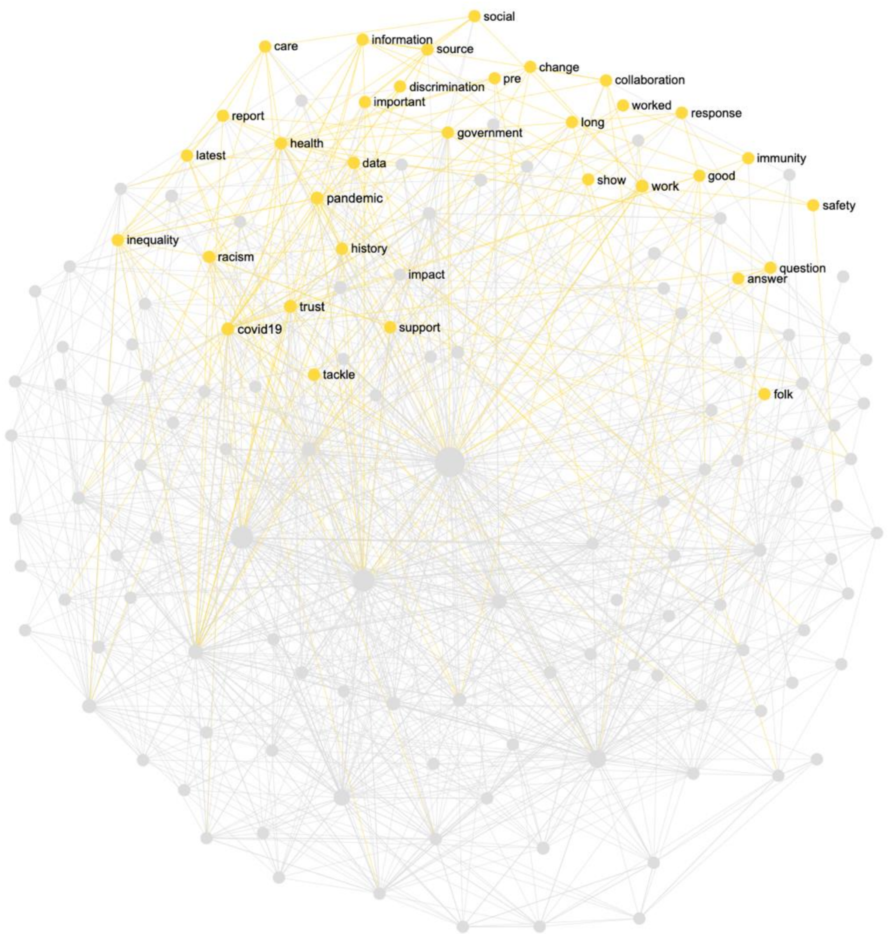

Cluster 2: Racism and health inequalities Keywords: “trust,” “pandemic” and “work.”

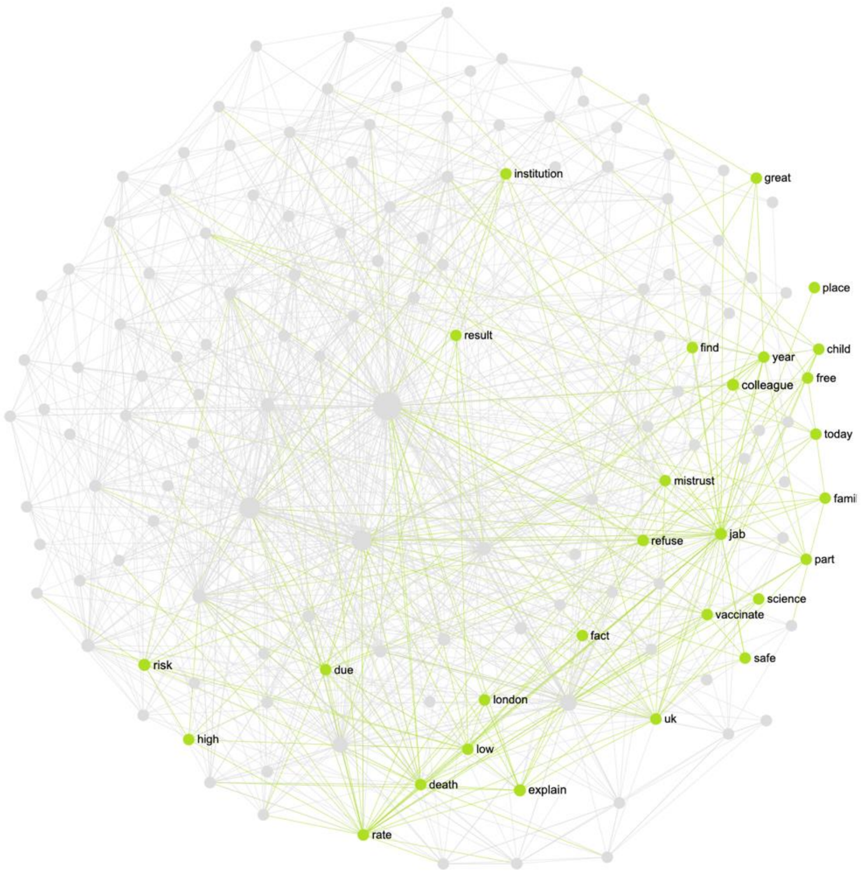

Cluster 3: Vaccine side effects Keywords: “jab,” “risk” and “explain”

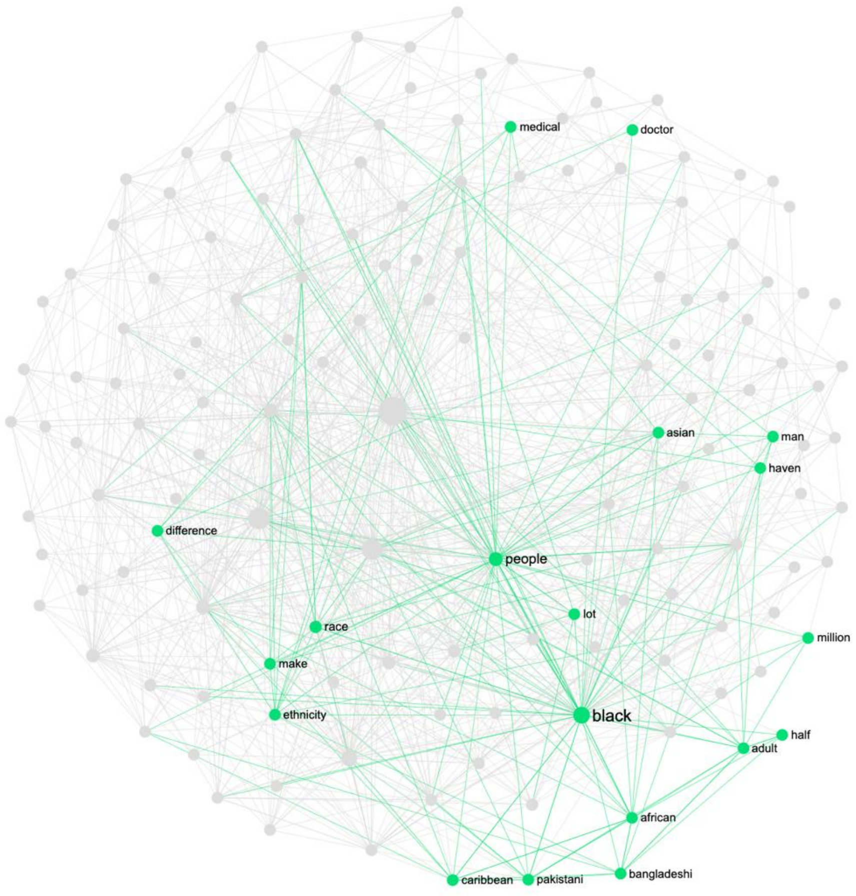

Cluster 4: Vaccine uptake in Black African and Caribbean communities Keywords: “black,” “people '' and “race”
