# Supplementary material for: A Snapshot of COVID-19 Vaccine Discourse Related to Ethnic Minority Communities in the United Kingdom Between January and April 2022: Mixed Methods Analysis
Source: JMIR Form Res. 2024 Mar 26;8:e51152. doi: 10.2196/51152 (PMC10968668; doi:10.2196/51152)
Supplement: Multimedia Appendix 4 [file formative_v8i1e51152_app4.pdf]

<u>Examples of conversation clusters in YouTube videos 1 and 3 related to themes of racism and mistrust in the UK</u>

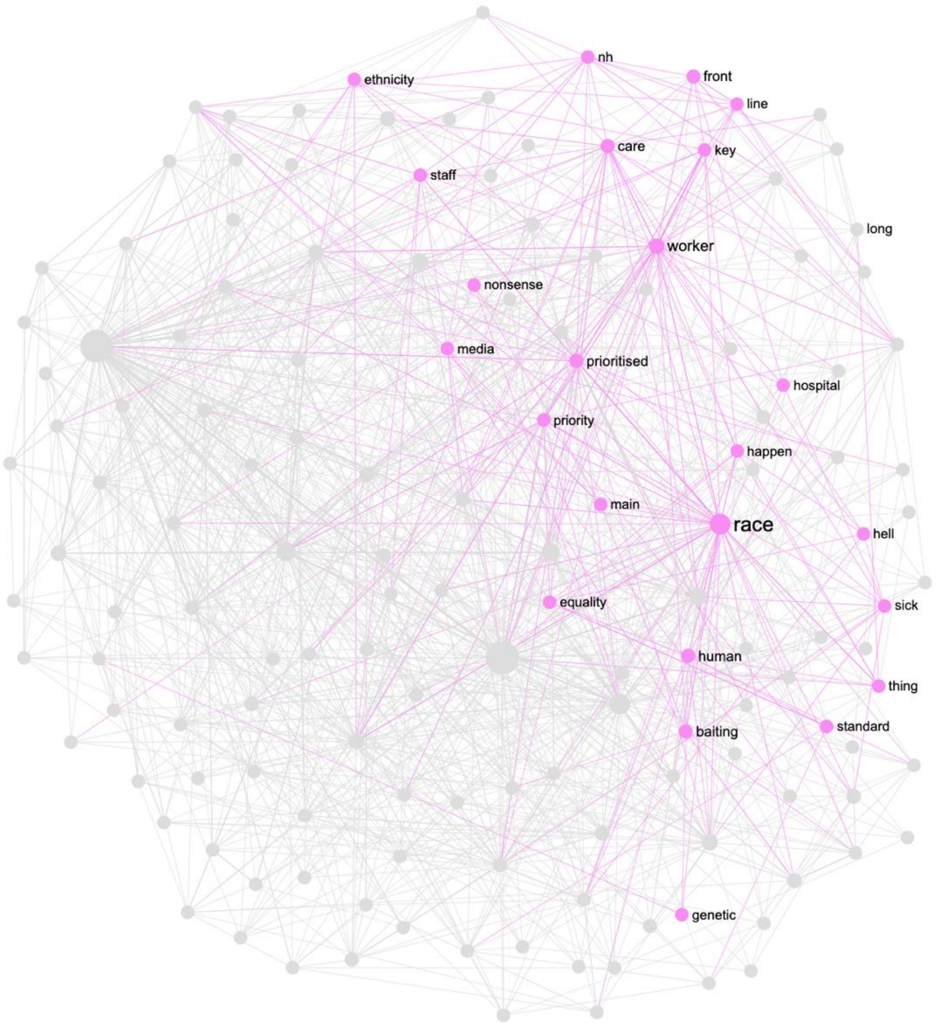

Video 1 example of cluster of conversation related to themes of racism. Keywords: Vaccine uptake in the community Keywords: ”race," ”worker," and ”baiting"

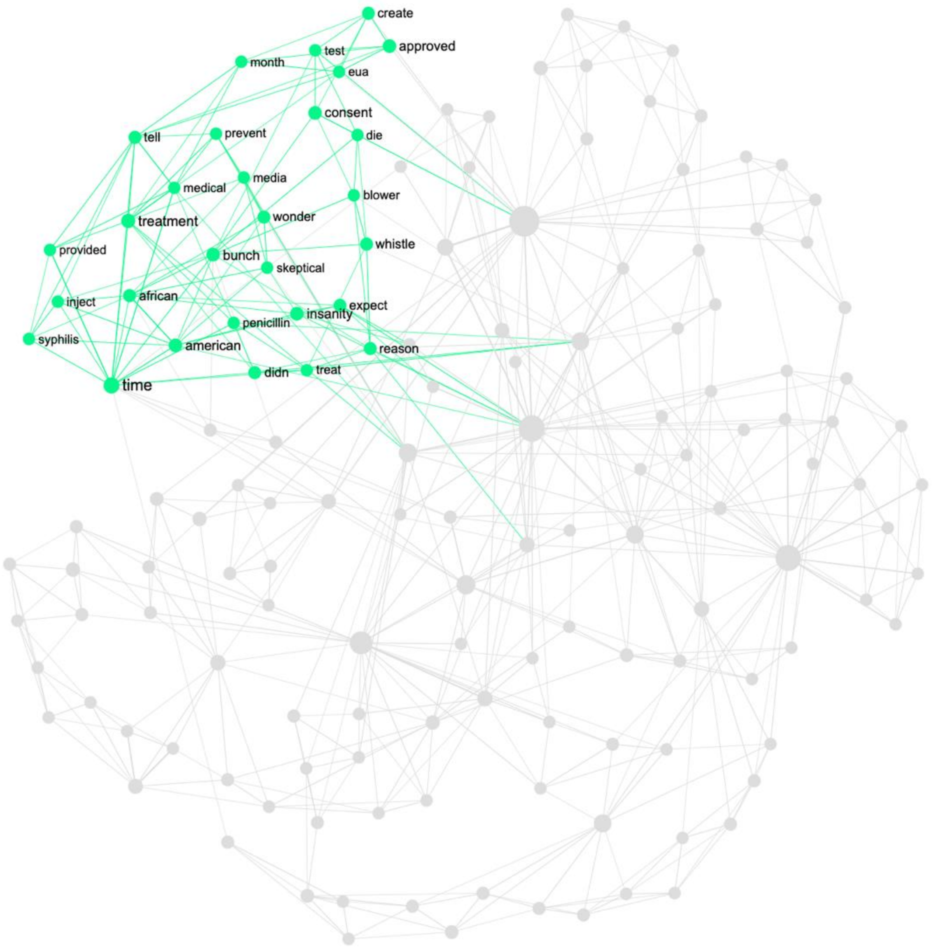

Video 3 example of cluster of conversation related to themes of mistrust. Keywords: Vaccine uptake in the community Keywords: ”time," ”treatment," and ”american"
